# Dissertation on the Effects of Carious Teeth

**Published:** 1852-10

**Authors:** Abr. Robertson

**Affiliations:** Manchester, N. H.


### ARTICLE XI.

*Dissertation on the Effects of Carious Teeth. Read before the American Society of Dental Surgeons, at their Annual Meeting, at Newport, R. I., August* 3, 1852. By ABR. ROBERTSON, D. D. S., of Manchester, N. H.

*Mr. President and Fellows of the*
*American Society of Dental Surgeons:—*

IN the dissertation, which, by your appointment, I shall read to you to-day, about all that I have proposed to myself to attempt, has been to say enough to elicit some attention to, and, if I may, to call forth some remarks, by the gentlemen of this society, upon a subject, which, although for some years past, I am aware has received a great deal of attention by many of our best dentists, and perhaps by all the members of this society, still seems to me to be deserving of far more attention than even now is usually given to it, as I believe, by dentists; and especially, by the medical profession; because I know that it is not an uncommon thing for patients who are suffering from irritable lungs, from "nervousness," from marasmus, or from other debility, to be recommended to the country for change of air—for purer air;—when they take with them—*are allowed to take with them*—a perfect cesspool of filth, containing matter both animal and vegetable, constantly fermenting and decomposing, and emitting nauseous gases, sufficient to cóntaminate all the air about them, (unlesss it be to their windward in a gale,) and through which all the air they breathe must pass. Because, I have frequent occasion to know that patients are often treated, by medication, for dyspepsia, for bronchitis and for phthisis pulmonalis, when the commencement of the alimentary canal, and of the trachea, is in a state of constant inflammation or irritation from the effect of diseased and decomposing teeth; the whole nervous system disturbed by the same cause; and every particle of food taken into their stomachs, and every breath of air taken into their lungs, vitiated and rendered unwholesome by noxious inhalations, or by the admixture of disordered and unhealthy saliva; and all this train of evils allowed to remain uncorrected—perhaps, unheeded. Because I hear a great many complain of being troubled with, and of having been treated for, "neuralgia," where there is obvious nervous irritation produced and constantly kept up by dead roots, or by badly diseased teeth; and with no attempt to remove the cause. Because I often see and hear of treatment by poultices, by epispastics and otherwise, for tumefactions of the face, (sometimes called—though entirely unmeaningly—"ague of the face,") without attempting to remove, or so much as to inquire into the cause. I say that it requires far more attention than, *as I believe*, is usually given to it, and especially by the medical profession, because I am not aware that the influences of diseased teeth upon the general health is scarcely alluded to in any of the works on medical practice, or in any of the lectures in any of the medical colleges. And because I have heard many physicians, and some of them eminent in their profession, say, "I know nothing about the diseases of the teeth!" Now all this might be, in some measure, excusable, if the teeth were isolated organs—if they had no connexion with any other part of the system. But it must be remembered, that although, in their chief substance, they have but a low degree of vitality—are not highly organized—still each tooth has at least one branch, and some as many as four or more distinct branches of nerve supplied to them; and that they are placed in that most important cavity, the mouth; forming, in part, and guarding the entrance to the alimentary canal and trachea; through which passages almost all substances of nutriment and of vivification are received. And that they must, therefore, both by sympathy and function, produce important influences upon the whole organization. Have I not then occasion to say, that, "as I believe," the connexion between the diseases of the general system with, and their dependence upon the diseases of the teeth, usually receives far less attention than the importance of the subject demands? And may their diseases and influences be overlooked or neglected by dentists, or by physicians with impunity? Let us see.

A physician is called to a patient laboring under severe fever; with hard, frequent pulse, restlessness, thirst, pain in the head, and intolerance of light. At the first glance he perceives that an eye is inflamed; and, on inquiry, finds that not many days before, a particle—a very minute particle—of sand or of metal has been blown or cast into and lodged upon that eye. The cause of all this great derangement of the whole system, this pain, this thirst, this fever, is at once explained. But does he commence treating those symptoms of general derangement of the system with antiphlogistics or refrigerants, without first removing the cause of that derangement, to wit: that minute particle that caused that *local* inflammation? Surely not; for every physician certainly knows that well established, that obvious first principle of surgery and of medicine, that to treat any disease successfully, he must first remove the cause.

The physician may, in like manner, be called to a patient who is in the utmost agony. His appearance frightful. Horror depicted in every feature. His eyes distended and bloodshot. His head thrown back, while his neck is drawn forward. The sterno-cleido-mastoideus muscles, by their rigidity, stand out prominent like broad thongs of raw-hide upon his neck. The muscles of his abdomen present to the hand the feel of boards beneath the skin. In a few hours, perhaps, death comes to relieve the sufferer—the scene is closed. But the physician's inquiries have revealed the fact, that his patient had, not long previously, stuck a little pin, or a small splinter of wood in his hand or his finger, or a nail in his foot. The matter is all satisfactorily explained now. A minute branch of some comparatively unimportant nerve in a remote part of the system has been wounded! The case is tetanus. Is it then unreasonable to suppose that a tooth so far decayed that its pulp is exposed and inflamed, may produce disturbance beyond the local seat of disease? And more especially, if that tooth is, or if many teeth are so far decayed that their whole crowns are gone; the vitality of their roots destroyed, but they still sticking in their sockets, or in the gum only, and producing inflammation and suppuration, little, if any less, than so many splinters of wood would do, may be the cause of general nervous irritation, of fever, of—death? That such is the fact—that diseased teeth do produce, directly or indirectly, almost all manner of diseases, and their ultimate consequence—death, numerous examples might be quoted to prove; but I shall not now make quotations. What is written "in the books" all can read. I intend only to refer to a few things that I have observed in my own practice, and I will not weary you with many.

Neuralgia would seem to be a very common, almost a fashionable disease. I have seen a great many cases—probably many hundreds—where patients have told me that they were, and for a long time had been, troubled with neuralgia; and many who had been treated for it by medication; but I do not now recollect but very few cases—perhaps not more than three or four—of facial neuralgia, where the cause could not be directly traced to diseased teeth, or dead roots of teeth; nor, where a proper treatment to restore them to health, or, if they were past such restoration, their removal would not cure their (very improperly I think) so called neuralgia.

I very well recollect the case of an old gentleman in Massachusetts, who, about two years ago, wished me to examine a lower bicuspid tooth that gave him a great deal of trouble. On examination I found that the alveolar process and gum had very much receded from the tooth; that it was, in consequence, dead and very loose, and producing a very considerable degree of inflammation. I advised its extraction; and with my fingers (for I had no instruments with me) extracted the tooth. Not long afterwards, the old gentleman sent me word that I had not only cured him of his sore and aching tooth, but also of a rheumatism in his arm, that, for a long time, he had been troubled with. We have quite a number of cases reported, in our journals and books on dentistry, of a similar kind; but as I am not aware the pathology of rheumatism is very well understood—that the conditions on which it depends are very fully known—I do not pretend to say that I think diseased teeth are a common cause of rheumatism, or, that they ever are a direct cause of that disease. I doubt indeed if they ever are. But this I do believe and say. If the system is predisposed to rheumatism, or almost any other disease, diseased teeth may, and probably often do, develop it. It is as well established a fact, perhaps, as almost any in the science of medicine, that individuals may be hereditarily predisposed to phthisis—be born, even, with tubercles in the lungs—but by carefully avoiding whatever might tend to develop the disease, as all depressing agents and circumstances, excessive excitements, and nervous irritations, they sometimes live to a good old age, and these tubercles remain dormant. On the other hand, the most careless observer knows, that, in those thus predisposed, numerous and even slight causes—as a little exposure to sudden changes in the atmosphere; a wetting of the feet; night watching; continued anxiety or grief; and the like, develop it, in all its fearfulness, and its almost certain result. That the nervous irritation from diseased teeth may do this, Dr. Bond, in his "Dental Medicine," quotes one or two very interesting cases, to show. A few weeks since, a friend of mine, now a dentist, related to me a case that came under his observation, some sixteen or eighteen years ago, that I think worth relating for its bearing on this point. It was a young lady, then of this state, (R. I.,) who, at the age of about twenty years, was supposed by her physician, her friends, and herself, to be far gone with consumption. About this time a young gentleman, who was a medical student, and who had paid some attention to dentistry, visited her father's. On being allowed to examine her mouth, he found her teeth in a very bad condition, and recommended that several of them be extracted, and some be filled. She, at first, objected, on the ground that, at the most, she could live but a very short time, and, therefore, she thought it unnecessary to submit to the pain of such an operation, when it could be of so little importance to her. Her friend, however, persuaded her to allow him to remove those that were past restoration. From that day she began to amend. Her health was soon entirely restored. She was afterwards married; and about a year ago, when my friend last heard of her, she still enjoyed good health. But, as I have said of rheumatism, I do not suppose that diseased teeth, directly, produce consumption. But that the irritation, and depression, and loss of rest, and of sleep caused by them, may, and probably often does develop this disease, I have no doubt.

But, without dwelling on diseases that are, or may be, *indirectly* produced, or developed by these causes, I shall venture to say that there are doubtless many, and serious diseases, produced *directly* by decayed teeth.

I have already alluded to a large amount of disease, of a very painful character, under the denomination of neuralgia, where this agency is most palpable, direct, unmistakable; and I scarcely need allude to the various tumors, some of them of the most malignant kinds, about the jaws, and the glands contiguous to them, caused by the irritation of decayed teeth. They are not of very unfrequent occurrence, nor of doubtful origin.

That distressing, and often fatal disease, bronchitis, there can scarcely be a doubt, is frequently produced, or at least, greatly aggravated, by inflammation extending from the gums to the fauces and throat, as well as by the debility occasioned by the disturbed sleep, the want of rest, and the impure air of which decayed teeth are the certain cause.

But there is another most annoying and troublesome disease, or perhaps I should rather say, class of diseases, not always very clearly defined or understood, but known by the common —the very popular—designation, dyspepsia, where the influences of decayed teeth are most direct in producing it. They are, in my belief, by far the most common cause of dyspepsia. Their influences here, too, act in several ways. Where an individual has many badly decayed teeth, the food is usually but slightly masticated. It is, therefore, not sufficiently disintegrated, nor properly mixed with saliva—it is not properly prepared for the stomach. It is indeed very badly prepared for the stomach; for the little saliva that is mixed with it is impure, vitiated. All the saliva, in fact, that passes into the stomach, whether with food or otherwise, is of an impure, vitiated, irritating character. The food is, therefore, imperfectly digested. Decay of the teeth, being, as I believe, and as I think is now generally believed by those who have given most attention to it, to be almost, if not altogether, simply a decomposition of their substance, we have, therefore, in such cases, the saliva constantly mixed with the decomposed and putrid animal matter. And where the use of the brush is neglected so that the teeth become loaded with tartar, as often happens, we have vegetable matter added to this decomposing, putrid mass of filth. How irritating! How nauseating! How disgusting! The gas arising from such decomposition, renders all the air taken into the lungs impure, and prevents the proper arterialization of the blood. That they constantly irritate the nervous system I need not here repeat. Thus marasmus is produced by badly digested food, by impure, improperly arterialized, blood, and by constant nervous irritation.

So fully do I believe dyspepsia to be a natural result of these causes, that I rarely, if ever, look into a patient's mouth containing many badly diseased teeth, without expecting to find that patient troubled, more or less, with dyspepsia; and my expectations are almost always verified by inquiry. I recollect a marked case of dyspepsia from decayed teeth, that, in my practice, came under my observation about three years ago; a sketch of which I will give you. A lady of about thirty years old—married, and the mother of two or three children—came to consult me about her teeth. She was then, and had been for some years, suffering badly from dyspepsia. Her complexion was sallow—almost of a cadaverous hue. She was feeble, and much emaciated. On examining her mouth, I found every tooth in her upper jaw—the whole sixteen—badly decayed, and the gums inflamed and turgid. I recommended the removal of all those teeth; and with her consent, proceeded to remove them all, which I accomplished at one sitting, of perhaps ten minutes. Her health, from that time, began to improve—her strength to increase—her complexion to brighten. She is now a robust healthy woman. Similar cases are by no means unfrequent; and to attempt the cure of such a case by change of scene, or of climate, by rest, by exercise, or by medication, without first removing that cause, would be very like filling a sieve with water—a hopeless and a tiresome task.

Before quitting, I must say a little more in relation to the miasmal phase of this subject.

Our legislators enact laws, and our municipal authorities appoint officers—"boards of health"—whose especial duty it is to see that no filth, and, particularly, that no animal or vegetable matters, be thrown upon, or left in the streets and alleys of our towns and cities, to decompose and produce miasmata; and that vaults and cess-pools be not left open to vitiate our air, and render it unhealthful. And all this very properly; and we freely pay our taxes for the making, and the executing such laws. The health of the community requires it. And shall the conservators of the health of the people—the physicians, or the dentists, then overlook or disregard this far more concentrated miasma, and its source—decayed and decaying teeth—situated as it is in the very gate way of life of their patients?

Although I may possibly place my estimate of the evil influences of decayed teeth, upon the general health of the system, too high; still, I think, that this, at least, must be admitted. The cause of a very large share of all the diseases to which the human system is subject, depends, in some way, either upon unwholesome food, (and I see very little difference in the result, whether it is so in itself, or is rendered so in the eating of it,) upon miasmata, or upon nervous irritation; and, therefore, many of them *may* be caused, or if not caused, *may* be developed by the vitiation of the food, by the miasma, or by the nervous irritation produced by dead and diseased teeth; and that it must be, at least, *safe* practice in the treatment of almost all, if not of all diseases;—and judicious prophylactic treatment as well—to remove, or allay all sources of nervous irritation, and of depression. And I will further hazard the opinion, that the teeth and their appendages are more frequently the seat of nervous irritation than any other portion, if not, than of all other portions of the organization; and that, if medical gentlemen when investigating the nature and the causes of the diseases that they are called to treat, will make it a point, carefully to examine the condition of the mouths, and particularly the teeth of their patients, they will there find a far more fruitful source of disease, and of its development, than very many of them, at least suppose.

It may be said that these remarks are calculated for physicians—for general practitioners—rather than for dentists. Be it so. I would speak to them on this subject. And I know of no place, from whence I can speak to them with more effect, or with better prospect of being heard, than in and through this Society. But if they—our elder brothers—overlook or disregard this subject, it is the more important that we, in our humbler sphere, should pay the more attention to it; that, although we may not be called upon to treat the diseases of which they are the cause, we may do, and be fully prepared to do, all in our power to prevent their occurrence.

### ARTICLE XII.

*Convulsions Cured by Lancing the Gums.* By V. M. SWAZYE, D. D. S.

